# Supplementary material for: Analysis of the Role of N-Linked Glycosylation in Cell Surface Expression, Function, and Binding Properties of SARS-CoV-2 Receptor ACE2
Source: Microbiol Spectr. 2021 Sep 8;9(2):e01199-21. doi: 10.1128/Spectrum.01199-21 (PMC8557876; doi:10.1128/Spectrum.01199-21)
Supplement: Reviewer comments [file reviewer-comments.pdf]

AMERICAN SOCIETY FOR MICROBIOLOGY | Microbiology Spectrum

Analysis of the Role of N-glycosylation in Cell-surface expression, Function and Binding Properties of SARS-CoV-2 receptor ACE2

Alberto Brandariz-Nuñez and Raymond Rowland

Corresponding Author(s): Alberto Brandariz-Nuñez, University of Illinois at Urbana Champaign

**Review Timeline:**	Submission Date:	August 6, 2021
	Accepted:	August 13, 2021

Editor: Ujjwal Neogi

Reviewer(s): The reviewers have opted to remain anonymous.

Transaction Report:

(Note: With the exception of the correction of typographical or spelling errors that could be a source of ambiguity, letters and reports are not edited. The original formatting of letters and referee reports may not be reflected in this compilation.)

DOI: https://doi.org/10.1128/Spectrum.01199-21

August 13, 2021

Prof. Alberto Brandariz-Nuñez
University of Illinois at Urbana Champaign
Pathobiology
112 west hill street
Champaign, IL 61820

Re: Spectrum01199-21 (Analysis of the Role of N-glycosylation in Cell-surface expression, Function and Binding Properties of SARS-CoV-2 receptor ACE2)

Dear Prof. Alberto Brandariz-Nuñez:

Your manuscript has been accepted, and I am forwarding it to the ASM Journals Department for publication. You will be notified when your proofs are ready to be viewed.

The ASM Journals program strives for constant improvement in our submission and publication process. Please tell us how we can improve your experience by taking this quick Author Survey.

As an open-access publication, Spectrum receives no financial support from paid subscriptions and depends on authors' prompt payment of publication fees as soon as their articles are accepted. You will be contacted separately about payment when the proofs are issued; please follow the instructions in that e-mail. Arrangements for payment must be made before your article is published. For a complete list of **Publication Fees**, including supplemental material costs, please visit our website.

Corresponding authors may join or renew ASM membership to obtain discounts on publication fees. Need to upgrade your membership level? Please contact Customer Service at Service@asmusa.org.

Thank you for submitting your paper to Spectrum.

Sincerely,

Ujjwal Neogi
Editor, Microbiology Spectrum

Journals Department
American Society for Microbiology
1752 N St., NW
Washington, DC 20036
E-mail: spectrum@asmusa.org
